# Integrative ceRNA network analysis in monozygotic twins reveals shared and disorder-specific molecular signatures in major psychiatric disorders

**DOI:** 10.1186/s12888-026-07894-5

**Published:** 2026-02-14

**Authors:** Chenglin Lu, Jianqiang Bi, Ruobing Liu, Jiazhuo Lan, Yang He, Chaoying Ni, Xiaohui Wu, Cunyou Zhao

**Affiliations:** 1 Key Laboratory of Mental Health of the Ministry of Education, Guangdong-Hong Kong-Macao Greater Bay Area Center for Brain Science and Brain-Inspired Intelligence, Guangdong-Hong Kong Joint Laboratory for Psychiatric Disorders, Guangdong Province Key Laboratory of Psychiatric Disorders, Guangdong Basic Research Center of Excellence for Integrated Traditional and Western Medicine for Qingzhi Diseases, Guangdong Mental Health Center, Guangdong Provincial People’s Hospital (Guangdong Academy of Medical Sciences), and Department of Medical Genetics, School of Basic Medical Sciences, Southern Medical University, Guangzhou, Guangdong 510515 China; 2 Experimental Education/Administration Center, School of Basic Medical Science, Southern Medical University, Guangzhou, China; 3 Department of Psychiatry, First Affiliated Hospital, Jinan University, Guangzhou, 510630 China; 4 Department of General Surgery IV, Yan’an Hospital of Kunming City, Kunming Medical University, Kunming, 650051 China

**Keywords:** Schizophrenia, Bipolar disorder, Major depressive disorder, MEGENA, Monozygotic twin pairs

## Abstract

**Background:**

Psychiatric disorders such as schizophrenia (SCZ), bipolar disorder (BD), and major depressive disorder (MDD) share overlapping features but arise from distinct molecular mechanisms. Competitive endogenous RNA (ceRNA) networks, where long non-coding RNAs (lncRNAs) and mRNAs compete for shared microRNAs (miRNAs), represent a key regulatory layer. This study sought to identify disorder-specific and convergent ceRNA regulatory signatures across these conditions.

**Method:**

We constructed an integrative analysis of whole-transcriptome and small RNA sequencing data from peripheral blood samples of monozygotic twin pairs discordant for disease. Differentially expressed mRNAs, lncRNAs, and miRNAs were identified within each discordant pair and integrated with co-expression modules from external transcriptomic datasets constructed by Multiscale Embedded Gene Co-expression Network Analysis (MEGENA). This enabled the construction of disorder-specific ceRNA networks and the identification of core regulatory components.

**Results:**

We identified ceRNA networks for each disorder, revealing 19 miRNAs shared across all three disorders, while lncRNAs and mRNAs were primarily disorder-specific. Among the shared miRNAs, hsa-miR-29a-3p was downregulated in both SCZ and MDD, regulating distinct ceRNA axes involving *COL6A6*. Functional enrichment analysis of hub ceRNA networks revealed the convergent involvement of extracellular matrix (ECM)-receptor interaction pathways. Notably, *COL6A6* (SCZ and MDD) and *ITGB8* (BD) were key components of these pathways. Validation using independent brain and blood transcriptomic datasets demonstrated strong predictive potential for *ITGB8* in the blood and prefrontal cortex for BD and SCZ, and moderate predictive potential for *COL6A6* in the blood and anterior cingulate gyrus for SCZ.

**Conclusions:**

This study identifies non-coding RNA–mediated regulatory networks implicated in the molecular etiology of psychiatric disorders. Our findings provide a foundation for precision diagnostics and targeted therapeutic strategies in psychiatry.

**Supplementary Information:**

The online version contains supplementary material available at 10.1186/s12888-026-07894-5.

## Introduction

Psychiatric disorders, including schizophrenia (SCZ), bipolar disorder (BD), and major depressive disorder (MDD), are complex, heterogeneous conditions with substantial public health burdens [1]. These disorders exhibit significant common symptom overlap and epidemiological comorbidity [2]. Although numerous genetic risk variants have been identified [3], the mechanisms by which these variants interact with environmental and epigenetic factors to confer disorder-specific risk remains largely unclear. Growing genomic and transcriptomic evidence also indicates that SCZ, BD, and MDD share substantial biological architecture, suggesting partially convergent molecular pathophysiology. A cross-disorder analysis therefore enables the distinction between shared regulatory signatures and disorder-specific alterations.

The transcriptome, capturing both coding and non-coding RNAs, offers a quantitative molecular interface between genetic predisposition and disease phenotype [4]. Notably, only ~ 2% of the human genome encodes proteins [5], whereas the majority is transcribed into non-coding RNAs (ncRNAs) with critical regulatory functions [6, 7]. Long non-coding RNAs (lncRNAs), the most abundant ncRNA class, are highly expressed in the brain and increase with organismal complexity [8–10]. They modulate neuroinflammation, synaptic plasticity, neuronal regeneration, and neurotransmission, implicating them in the pathophysiology of neuropsychiatric disorders [11–15]. Similarly, microRNAs (miRNAs) – short (∼22 nt) post-transcriptional regulators – shape neurodevelopment and synaptic function [16–18]. Dysregulation of both lncRNAs and miRNAs has been consistently reported in blood and brain tissues from patients with SCZ, BD, and MDD, underscoring ncRNA-mediated regulation as an important layer of molecular pathology.

Given the central role of ncRNAs in brain function and psychiatric disease, the competitive endogenous RNA (ceRNA) framework offers a conceptually relevant and mechanistically informative approach for exploring transcriptomic dysregulation. In this model, lncRNAs act as molecular “sponges” that bind and sequester miRNAs, thereby modulating the expression of miRNA-targeted mRNAs [19, 20]. By integrating lncRNA-miRNA-mRNA interactions, ceRNA networks capture coordinated post-transcriptional regulation that may better reflect the subtle, polygenic, and modular architecture characteristic of psychiatric disorders. Recent studies have increasing applied ceRNA network analyses in psychiatric transcriptomic research [21–24], demonstrating their utility in identifying dysregulated regulatory modules and potential biomarkers. Building upon this foundation, our study aims to delineate both disorder-specific and convergent ceRNA-mediated regulatory signatures across psychiatric disorders.

Monozygotic (MZ) twin pairs discordant for psychiatric disorders, where one twin is affected and other remains unaffected, offer a powerful natural model for investigating disease mechanisms. As MZ twins share virtually identical genome [25] and are matched for key confounding variables (e.g., age, sex, family background), within-pair differences in gene expression can be attributed primarily to disease status [26]. This design minimizes the impact of genetic and shared environmental factors, making it particularly valuable for studying epigenetic regulation, including non-coding RNA-mediated effects.

In this study, we integrated whole-transcriptome and small RNA sequencing data from discordant MZ twin pairs with multiple external validation datasets. We applied Multiscale Embedded Gene Co-expression Network Analysis (MEGENA) [27] to construct multi-layered co-expression modules and identify lncRNA-miRNA-mRNA ceRNA network across SCZ, BD, and MDD. We further assessed their biological relevance of these regulatory interactions across five brain regions. This integrative framework enables the characterization of both shared and disorder-specific regulatory mechanisms and highlights potential RNA-based biomarkers for major psychiatric disorders.

## Materials and methods

### Human participants

The primary discovery cohort consisted of 12 MZ twin pairs discordant for psychiatric disorders, including five BD-discordant pairs (BDC), three MDD-discordant pairs (MDC), and four SCZ-discordant pairs (SDC; Supplementary Table S1). All twins were reared together from childhood and shared comparable early-life environments. Peripheral blood was collected for whole-transcriptome (RNA-seq) and small RNA (sRNA-seq) profiling.

To ensure methodological independence and avoid circular inference, co-expression networks were constructed using three external blood case–control datasets not used for differential expression analysis or biomarker testing (GSE46449: 49 BD/39 controls; GSE98793: 64 MDD/64 controls; GSE27383: 43 SCZ/29 controls). Biological relevance of candidate genes was further evaluated using three additional independent blood transcriptomic datasets (GSE124326: 61 BD/170 controls; GSE247998: 73 MDD/27 controls; GSE263180: 9 SCZ/20 controls), which were used exclusively for Receiver Operating Characteristic (ROC)-based validation. Cross-tissue consistency was assessed using two independent brain transcriptomic datasets (GSE53987 and GSE80655), encompassing diagnostic groups across dorsolateral prefrontal cortex, anterior cingulate cortex, and temporal cortex. No dataset contributed to more than one analytic step.

Zygosity was confirmed using the Qiagen Investigator Argus X-12 QS Kit (USA). All affected individuals met the diagnostic criteria of SCZ, MDD, or BPD according to the Diagnostic and Statistical Manual of Mental Disorder, Fifth Edition (DSM-5; American Psychiatric Association). Written informed consent was obtained from all participants after a full explanation of the study procedures. Peripheral blood samples were collected from all participants. Total RNA was isolated using standard phenol-chloroform method for RNA-sequencing (RNA-seq) and small RNA sequencing (sRNA-seq).

### RNA-seq or sRNA-seq processing

RNA-seq and sRNA-seq libraries were prepared and sequenced on the Illumina NovaSeq platform by BGI Solutions and Novogene. Raw sequencing quality was assessed using FASTQC (v0.12.1) and multiqc (v1.30). For sRNA-seq, adapter trimming and quality filtering were conducted using Trim Galore (v0.6.10) with the parameters “--quality 30; --length 10 and --max_length 30”. MultiQc quality-control reports for RNA-seq and sRNA-seq are provided in Supplementary Figure S1. Clean reads were aligned to the Homo sapiens reference genome (UCSC hg38) using miRDeep2 (v2.0.1.3) [28]. Known microRNA (miRNA) were quantified with reference to miRBase version 21 [29], and raw count were obtained through miRDeep2.

For RNA-seq, high-quality reads were aligned to the Homo sapiens reference genome (UCSC hg38) using Hisat2 (v2.2.1) [30] with the strand-specific parameter “--rna-strandness FR.” Unique mapped reads were filtered using SAMtools (v1.22.1) [31] to exclude secondary and non-primary alignments. Gene-level expression quantification was performed using StringTie (v3.0.0) [32] with default parameters, guided by the UCSC hg38 gene annotation (GTF file).

### Differential expression analysis and enrichment

Differential expression analysis (DEA) was conducted in R (v4.3.1). Prior to analysis, lowly expressed RNAs or miRNAs were filtered if more than 50% of the samples contained zero counts for a given transcript. To address potential batch effects arising from sequencing BD and MDD samples at two different facilities (Novogene and BGI), count data were adjusted using the ComBat_seq function from the SVA package (v3.54.0) [33]. For the SCZ cohort, all samples were submitted to Novogene during the same sequencing period and processed using consistent library preparation workflows and the same platform. As there was no temporal or procedural separation within the SCZ submissions, no additional batch correction was applied for this dataset. PCA plots before and after batch adjustment are provided in Supplementary Figures S2–S3.

Using the raw counts matrix, DEA was conducted for protein-coding RNAs (DE-mRNAs), miRNAs (DE-miRNAs), and lncRNAs (DE-lncRNAs) between affected and unaffected individuals within each discordant pairs using DESeq2 (v1.46.0) [34], implemented with a paired design to account for the monozygotic twin structure of the dataset. For each disorder, two variables were defined: (i) “group”, indicating disease versus control status within each twin pair, and (ii) “pair”, representing the family identifier for each MZ twin pair. The design formula used for constructing the DESeqDataSet object was: design = ~ pair + group. In this formulation, “pair” functions as a blocking factor that models the shared genetic background and within-pair correlation, while “group” captures the effect of disease status. For example, in the MDD cohort with three twin pairs, the pair variable was encoded as 1, 1, 2, 2, 3, 3, where matching numbers denote members of the same MZ twin pair. This approach ensures that differential expression is estimated within pairs, thereby controlling for genetic and shared early-life environmental factors.

Significant differentially expressed transcripts were defined by a p-value < 0.05 and an absolute log2 fold change (|log2FC|) > 0.585. This nominal threshold was used because psychiatric disorders typically exhibit subtle, small-effect transcriptional changes; applying stringent FDR correction at the discovery stage would eliminate the vast majority of potentially meaningful signals, as shown in our preliminary analyses. For DE-mRNAs and DE-lncRNAs, functional enrichment analysis was performed using Gene Ontology (GO) and Kyoto Encyclopedia of Genes and Genomes (KEGG) pathways via Metascape (v.5.20250701). Enriched terms were considered significant if they met the criteria of *p* < 0.05 and a minimum gene overlap > 3. Similar to the DEA rationale, nominal thresholds were adopted for enrichment analyses to avoid loss of biologically relevant pathways driven by small-effect genes, while full FDR-adjusted results are provided in the Supplementary Tables for transparency.

### Co-expression network analysis using public datasets

Public transcriptomic datasets (GSE46449, GSE98793, GSE27383) were obtained from GEO. Genes with zero expression in more than 50% of samples were removed. Batch effects in GSE98793 and GSE46449 were corrected using the empiricalBayesLM function in WGCNA (v1.73) [35].

Multiscale co-expression networks were constructed using MEGENA (v1.3.7) [27] (Supplementary Fig. S4). Briefly, pairwise gene–gene correlations were computed using Pearson coefficients, and significant correlation (FDR < 0.05) were used to construct a planar filtered network (PFN), retaining non-redundant edges. The PFN was decomposed into hierarchical modules via multiscale clustering, and modules were evaluated using compactness and hub significance thresholds (mod.pval < 0.05; hub.pval < 0.05). Modules containing ≥ 10 genes were retained.

Module–trait associations were assessed by correlating module eigengenes with diagnostic status. Modules with *r* > 0.2 and *p* < 0.05 were considered significantly disease-associated. Genes from significant modules were extracted and intersected with DE-mRNAs and DE-lncRNAs to obtain candidate transcripts for ceRNA construction.

### ceRNA network construction

Candidate DE-lncRNA‒miRNA interactions were obtained from the starBase database (v2.0, https://rnasysu.com/encori/), with user-defined parameters allowing exclusion of CLIP-seq-supported interactions. Candidate mRNA‒miRNA interactions were retrieved from TargetScan (v8.0) [36] and miRDB (v6.0) [37]; mRNA-miRNA pairs present in both datasets were kept to reduce false-positive predictions.

For each disorder, overlapping miRNA shared between DE-lncRNA‒miRNA and DE-mRNA‒miRNA interaction sets were identified. These miRNAs were further filtered by shared seeding-sequence binding sites across lncRNAs and miRNAs, ensuring that retained triplets reflected biologically plausible MRE-based competition.

Initial ceRNA networks were constructed for each discovery cohort and visualized in Cytoscape (v3.10.2) [38]. Each network was composed of three differentially expressed components: DE-mRNAs, DE-lncRNAs, and their common interacting miRNAs.

To identify core regulatory elements with higher disease relevance, we refined the ceRNA network by integrating DE-miRNAs. Only ceRNA triplets involving DE-miRNAs were retained, yielding a “hub” ceRNA network representing ceRNA interactions driven by miRNAs that were significantly dysregulated in the corresponding disorder. This step enhances disease specificity while avoiding over-stringent expression-based filtering that may remove true biological interactions in small twin cohorts.

### Shared molecular profiling and blood-brain biological relevance nomogram

To evaluate the biological relevance of DE-mRNAs and DE-lncRNAs identified in peripheral blood-derived ceRNA networks, we incorporated three independent blood datasets (GSE124326, GSE247998, GSE263180) and two brain datasets (GSE53987, GSE80655; Supplementary Table S1) for ROC curve analyses using the multipleROC R package (v0.1.1).

Candidate RNAs were selected based on (i) consistent differential expression across SCZ, BD, and MDD and (ii) presence in both the ceRNA network and blood and brain datasets. For each candidate gene, we fitted a univariate logistic regression model (group ~ gene expression) without additional covariates, as no clinical or medication variables were consistently available across datasets. Group differences were evaluated using a Wilcoxon signed-rank test, which we used in place of the default test implemented in multipleROC. Cross-validation or permutation was not applied, and this has been noted to avoid overstating predictive performance.

For each gene-region combination, we reported the AUC, 95% confidence interval, and exact P-value. A candidate was considered to exhibit biologically meaningful relevance if AUC > 0.65 and *p* < 0.05. Because analyses were restricted to a small, predefined set of candidate genes for independent validation rather than discovery, multiple-testing correction was not applied.

## Result

### Differential expression profiling in MZ twins discordant for psychiatric disorders

**Fig. 1:**
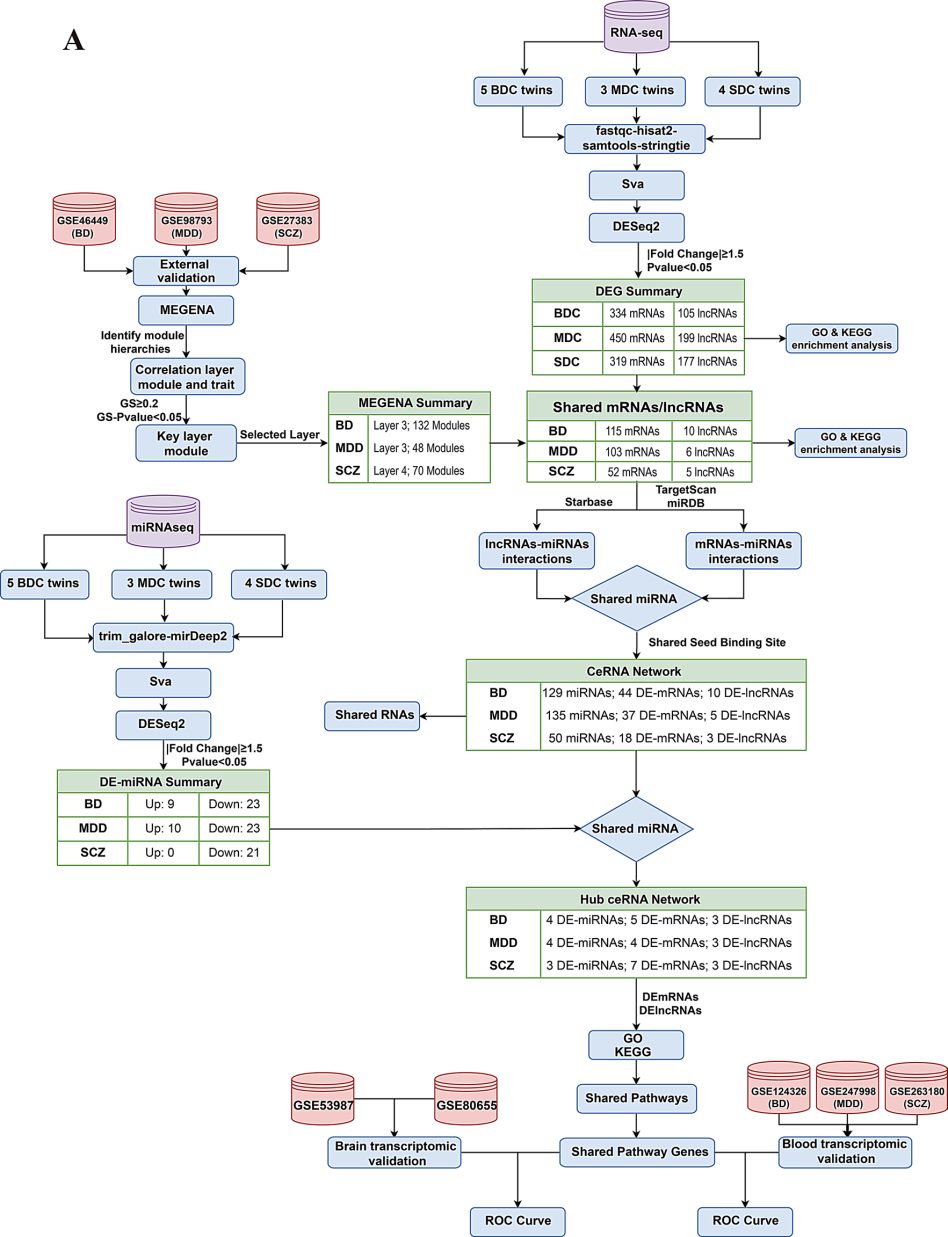

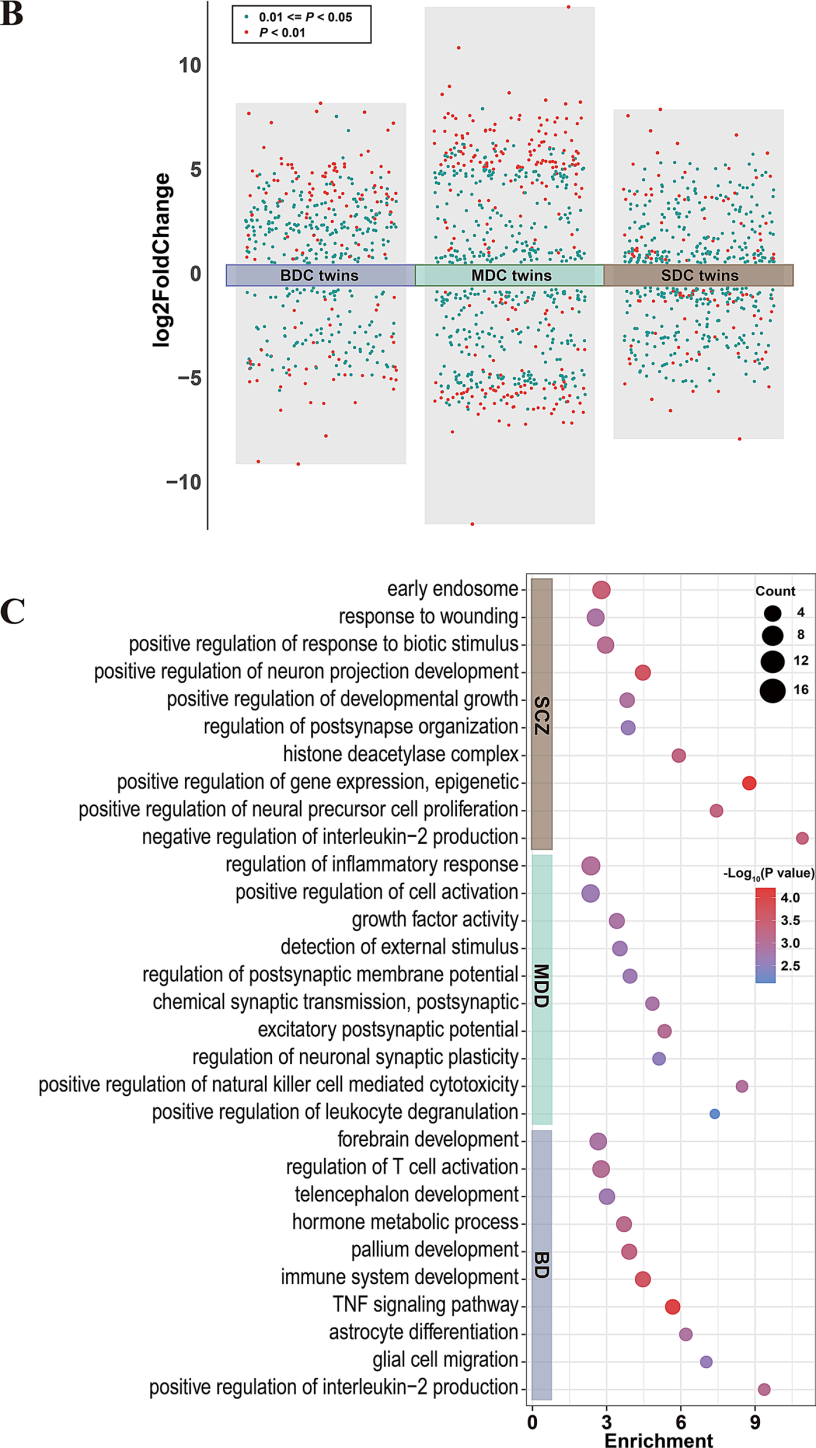
Overview of differential expression and enrichment analyses in monozygotic twins discordant for psychiatric disorders. (**A**) Flowchart illustrating the comprehensive analytical pipeline employed in this study. (**B**) Differential expression patterns of mRNAs and lncRNAs across MZ twins discordant for BD (BDC twins), MDD (MDC twins), and SCZ (SDC twins). Red dots represent significant differentially expressed (DE) RNAs (p-values < 0.01), while turquoise dots indicate RNAs with p-values between 0.01 and 0.05. (**C**) Dot plots of enriched Gene Ontology (GO) terms, including biological process (GO-BP), cellular component (GO-CC) and molecular function (GO-MF), and kyoto encyclopedia of genes and genomes (KEGG) pathways associated with the DE-RNAs in BD, MDD and SCZ. Only terms with p-value < 0.05 are displayed

An overview of the study design is presented in Fig. 1A. We performed whole-transcriptome DEA in MZ twin pairs discordant for psychiatric disorder. In the BDC cohort, we identified 439 differentially expressed genes (DEG; 334 DE-mRNAs, 105 DE-lncRNAs). The MDC cohort yielded 649 DEGs (450 DE-mRNAs, 199 DE-lncRNAs). In the SDC group, 496 DEGs (319 DE-mRNAs, 177 DE-lncRNAs) were detected (Fig. 1B, Supplementary Table S2-S4).

To investigate the functional relevance, we performed Gene Ontology (GO) enrichment analysis of these DEGs. In BD, DEGs were enriched in processes related to brain development and astrocyte function, such as cerebral cortex development, forebrain development, and astrocyte differentiation (Fig. 1C, Supplementary Table S5). In contrast, MDD- and SCZ-associated DEGs showed enrichment in synaptic function. Specially, MDD was associated with regulation of neurotransmitter receptor localization to the postsynaptic specialization membrane and excitatory postsynaptic potential, while SCZ was enriched for synaptic vesicle transport and regulation of neuronal synaptic plasticity. In SCZ, additional enrichment was observed in neuronal cell-related processes.

KEGG pathway analysis further highlighted disorder-specific regulatory landscapes. In BD, DEGs were enriched in the NF-kappa B, MAPK, and TGF-beta signaling pathways (Supplementary Table S5). In SCZ, calcium signaling and spinocerebellar ataxia pathways were prominent, along with genes implicated in neurodegenerative diseases. In contrast, MDD-associated transcripts were primarily enriched in metabolism-related pathways, including nucleotide metabolism and drug metabolism involving other enzymes.

### Multiscale gene co-expression network construction in psychiatric disorders

To elucidate disease-associated mRNA-lncRNA interactions, we applied MEGENA to external transcriptomic datasets for BD (GSE46449), MDD (GSE98793), and SCZ (GSE27383) from the GEO database (Fig. 1A and Supplementary Table S1). MEGENA identified six hierarchical layer of co-expression modules in the BD and seven layers in MDD datasets, and eight layers in SCZ. For the third layer of BD and MDD, there are 303 modules in BD and 164 modules in MDD, and 189 modules in layer 4 in SCZ. MEGENA identified seven hierarchical layer of co-expression modules in the BD and MDD datasets, and eight layers in SCZ. Module-disease associations were evaluate using Pearson correlation analysis (*p* < 0.05), retaining modules with correlation coefficients > 0.2 for further analysis. Significant modules from Layer 3 were selected in BD (132 modules) and MDD (48 modules), while Layer 4 modules were prioritized in SCZ (70 modules) (Supplementary Table S6).

**Fig. 2:**
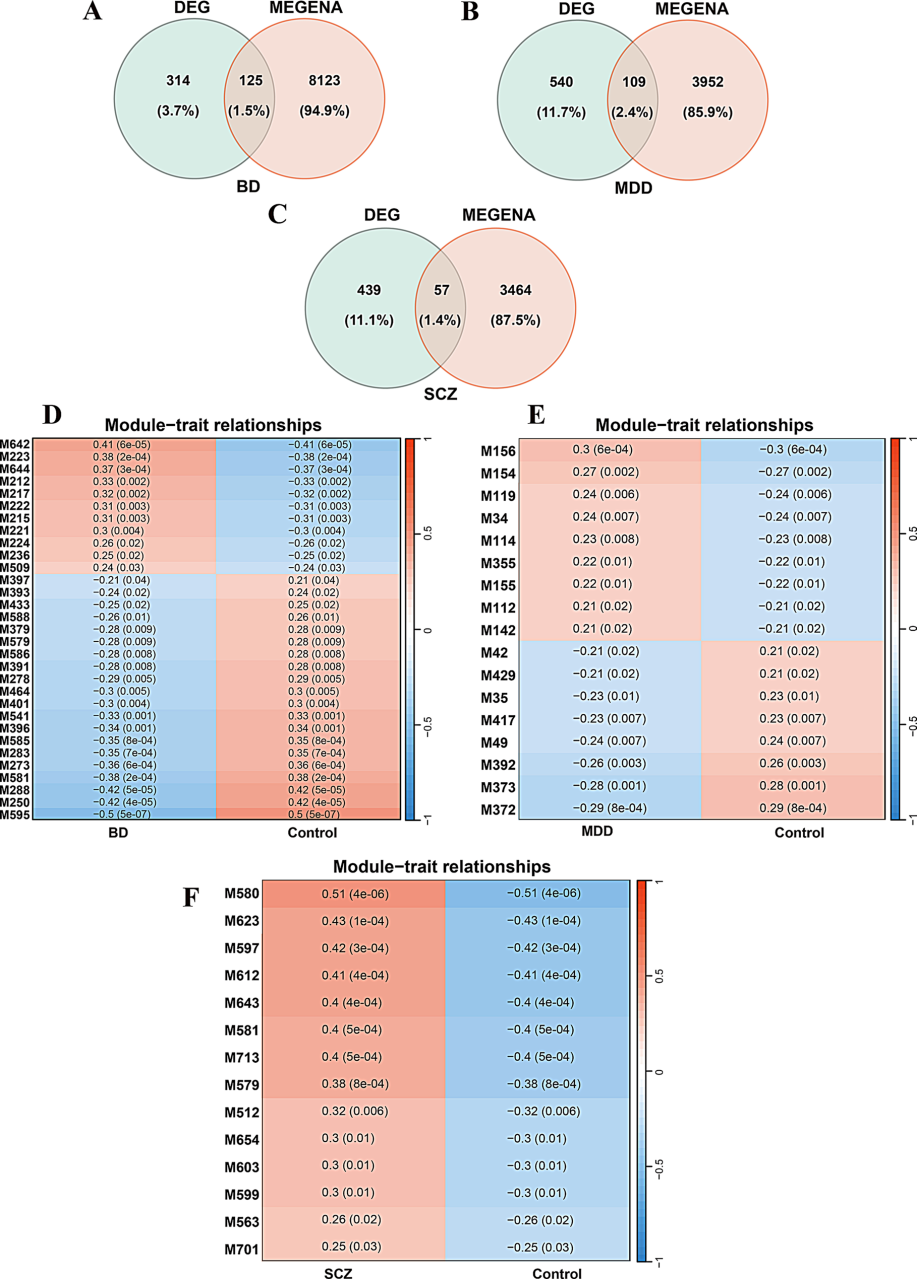
Intersection of DEGs and MEGENA modules, and modules-trait relationships in psychiatric disorders. (**A-C**) Venn diagrams illustrating intersections between DEGs (including mRNAs and lncRNAs) identified from MZ twin analyses and candidate genes from MEGENA modules derived from external datasets for BD (**A**), MDD (**B**), and SCZ (**C**). Numbers indicate the count and percentage of overlapping and unique genes within each dataset. (**D-F**) Heatmaps showing correlations between MEGENA modules containing intersecting candidate genes and disease status (BD, MDD, SCZ) compared to controls. Each row represents a module, and each column represents a phenotype (disease or control). The color scale reflects correlation strength: red indicates positive correlation, and blue indicates negative correlation. Numeric values represent correlation coefficients with corresponding p-values (shown in parentheses) for significant associations (*p* < 0.05)

To identify candidate components of ceRNA networks, were intersected these disease-associated MEGENA modules with DE-mRNAs and DE-lncRNAs from our twin cohort analysis. This integration yielded 125 candidate DEGs in BD, including 115 DE-mRNAs and 10 DE-lncRNAs; 109 DEGs in MDD, including 103 DE-mRNAs and 6 DE-lncRNAs; and 57 DEGs in SCZ, including 52 DE-mRNAs and 5 DE-lncRNAs (Fig. 2A-C, Supplementary Table S7). Modules containing these candidates were subsequently ranked by the number of overlapping transcripts, and those containing at least two candidate RNAs were visualized using heatmaps to depict their correlation with disease status (Fig. 2D-F).

To investigate the functional relevance of these candidate RNAs, we performed GO and KEGG enrichment analyses. In BD, the candidate RNAs were significantly enriched in processes related to pallium development and glial cell migration. In MDD, enrichment was observed in immune-related biological processes, including the positive regulation of natural killer cell and T cell mediated cytotoxicity. In SCZ, enriched terms included regulation of neurogenesis and forebrain development (Supplementary Table S8).

### ceRNA network construction and shared molecular profiling in psychiatric disorder

**Fig. 3:**
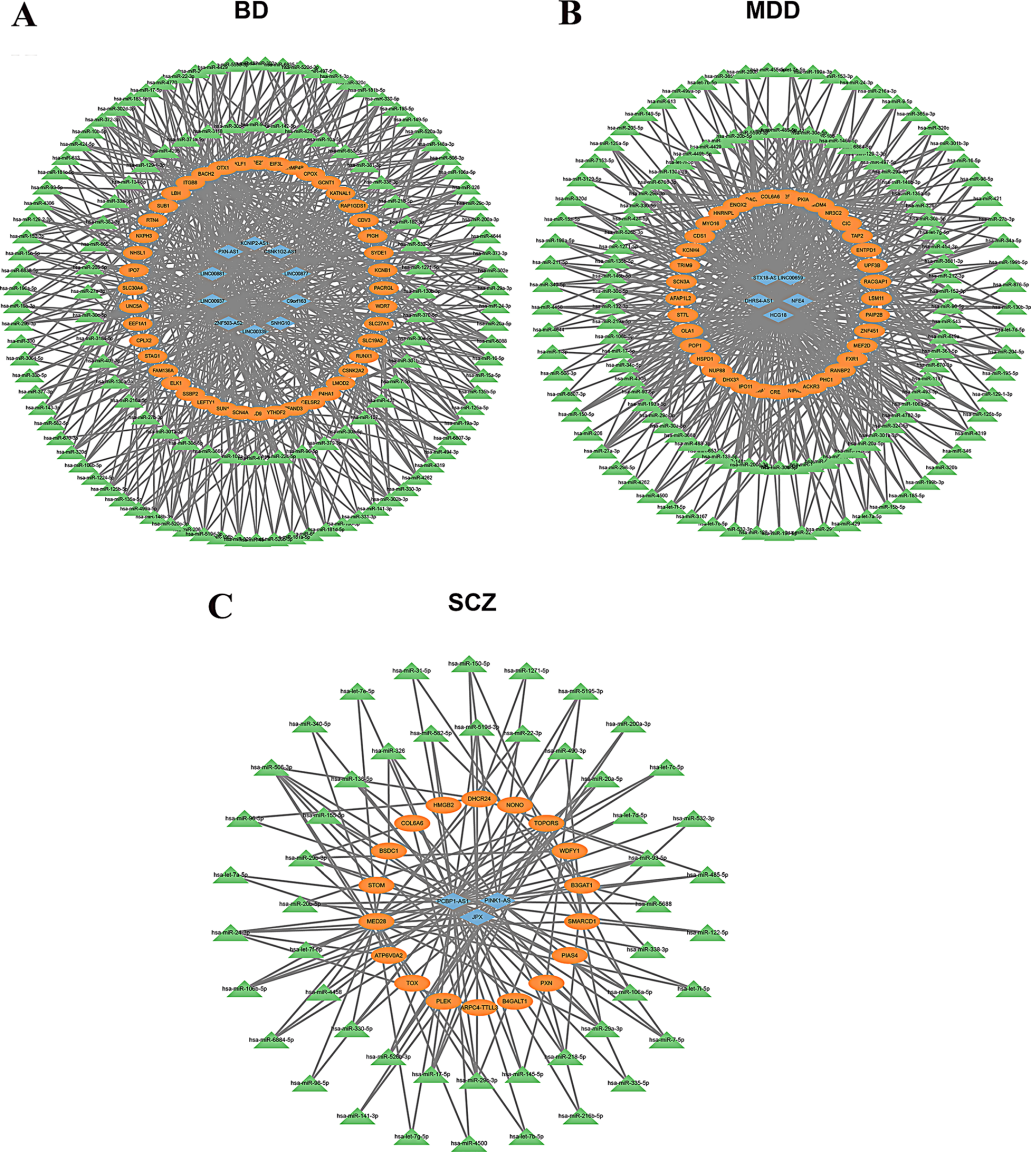

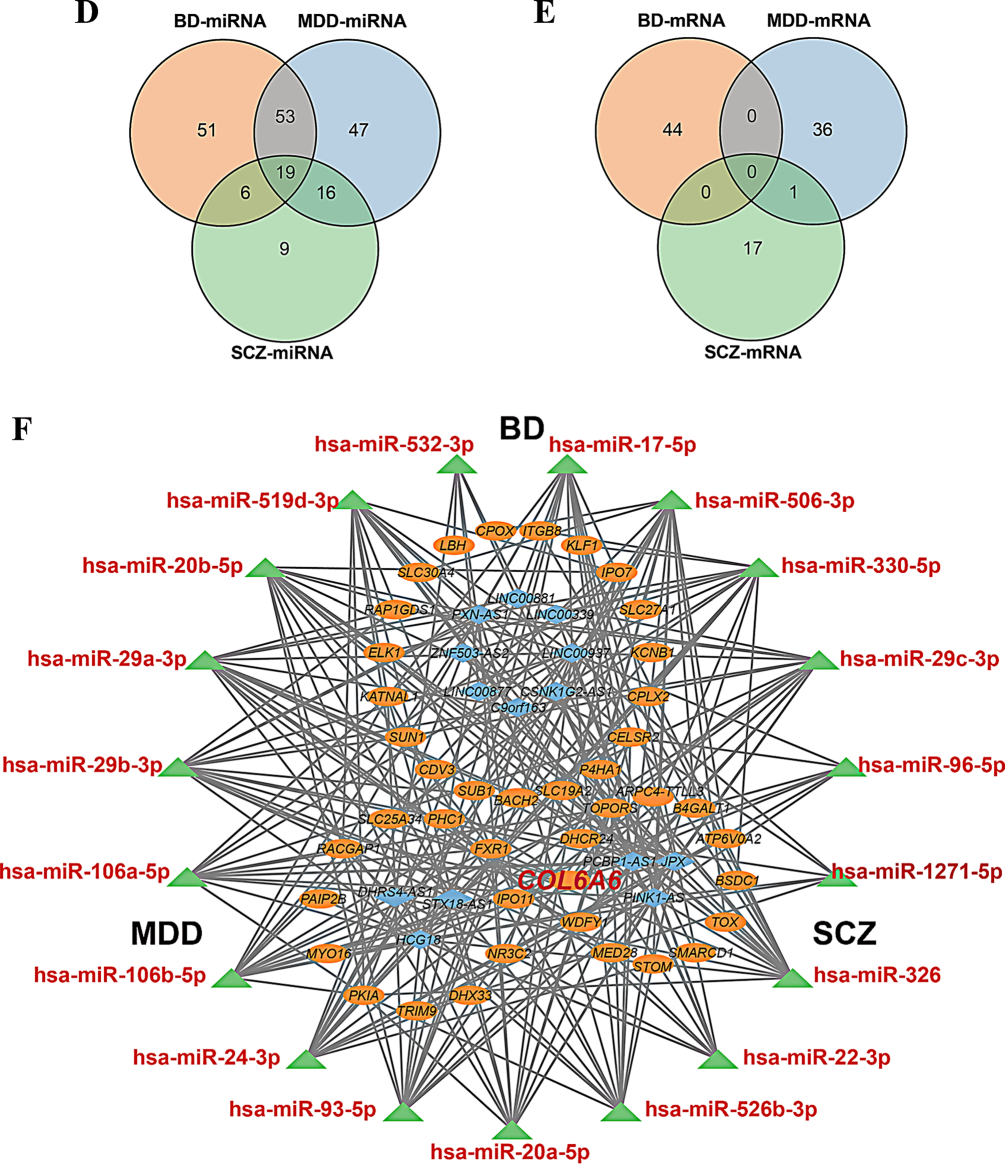
Construction of disorder-specific and shared ceRNA networks in psychiatric disorders. (**A–C**) Disorder-specific ceRNA networks constructed for BD (**A**), MDD (**B**), and SCZ (**C**). Each network depicts predicted regulatory interactions among lncRNAs (blue diamonds), miRNAs (green triangles), and mRNAs (orange ovals). Edges (gray lines) represent computationally predicted miRNA–lncRNA and miRNA–mRNA interactions derived from starBase, TargetScan, and miRDB databases. (**D**,** E**) Venn diagram illustrating the intersections of miRNAs (**D**) and mRNAs (**E**) within the ceRNA networks across BD, MDD, and SCZ. (**F**) Shared miRNA-centered ceRNA subnetwork across the three disorders, showing overlapping regulatory components. Despite no shared lncRNAs or mRNAs across all conditions, 19 miRNAs were identified as common regulators, forming a unified cross-disorder ceRNA network

To construct disorder-specific ceRNA networks, we first identified candidate mRNA-miRNA interaction pairs by intersecting prediction from TargetScan and miRDB, and identified candidate lncRNA-miRNA interactions using starBase (Fig. 1A). These interactions were used to construct ceRNA networks for each psychiatric disorder, which were subsequently visualized using Cytoscape. The resulting BD network included 129 miRNAs, 44 DE-mRNAs, and 10 DE-lncRNAs (Fig. 3A). The MDD network comprised 135 miRNAs, 37 DE-mRNAs, and 5 DE-lncRNAs (Fig. 3B), while the SCZ network consisted of 50 miRNAs, 18 DE-mRNAs, and 3 DE-lncRNAs (Fig. 3C, Supplementary Table S9).

To identify shared regulatory features, we compared the ceRNA networks across the three disorders.

Pairwise comparisons revealed further overlaps: 72 miRNAs were shared between BD and MDD, 25 between BD and SCZ, and 35 between MDD and SCZ (Fig. 3D). Notably, *COL6A6* emerged as the only DE-mRNA overlapped (Fig. 3E) between MDD (log_2_FC = -1.146, *p* = 4.79E-2) and SCZ (log_2_FC = 0.806, *p* = 4.97E-2). Nineteen miRNAs were present in all three disorder-specific networks (Fig. 3F), although their directions of differential expression were not necessarily consistent across disorders. Several of these miRNAs have been previously implicated in psychiatric pathophysiology [39–45]. These overlapping miRNAs were visualized in an integrated cross-disorder subnetwork using Cytoscape. In contrast, no DE-mRNAs or DE-lncRNAs were shared across all three disorders, suggesting that while certain miRNAs may serve as shared regulatory hubs, the mRNA and lncRNA components of the ceRNA networks are largely disease-specific. Despite limited overlap in mRNA and lncRNA, the consistent presence of shared miRNA suggests a degree of convergent post-transcriptional regulation among the disorders. In contrast, the disorder-specific lncRNA and mRNA components likely reflect the distinct molecular mechanism sand pathophysiological features of each condition (Supplementary Table S9).

### Disease-associated ceRNA network construction in MZ twins

**Fig. 4:**
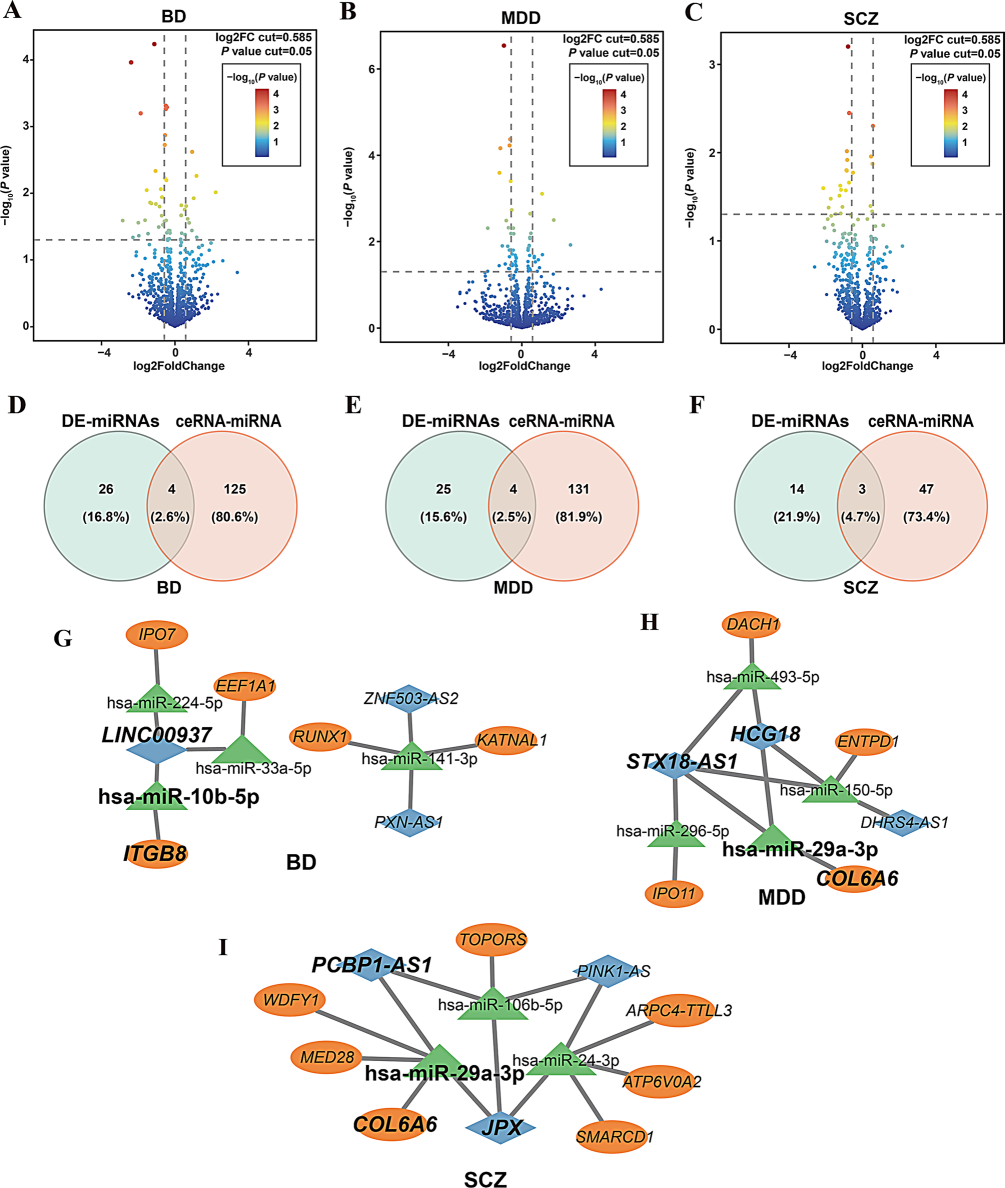
Integration of DE-miRNAs into disorder-specific ceRNA networks in psychiatric disorders. (**A-C**) Volcano plots displaying the distribution of DE-miRNAs in BD (**A**), MDD (**B**), and SCZ (**C**). The vertical dashed lines represent log2 fold change thresholds of ± 0.585, and the horizontal dashed line indicates the significance threshold at *p* = 0.05. Each point corresponds to a miRNA, color-coded by –log10(p-value). Significantly upregulated or downregulated miRNAs appear in warmer colors (red/orange), while non-significant miRNAs are in blue. (**D–F**) Venn diagrams showing the overlap between DE-miRNAs identified in the MZ twin cohorts (DE-miRNAs, green) and miRNAs included in the previously constructed disorder-specific ceRNA networks (ceRNA-miRNA, red) for BD (**D**), MDD (**E**), and SCZ (**F**). The intersecting miRNAs represent candidates incorporated into refined hub ceRNA networks. (**G–I**) Hub ceRNA subnetworks constructed for BD (**G**), MDD (**H**), and SCZ (**I**), based on DE-miRNAs shared with each disorder’s ceRNA network. Networks display regulatory interactions among DE-miRNAs (green triangles), DE-lncRNAs (blue diamonds), and DE-mRNAs (orange ovals). Edges represent predicted miRNA–lncRNA and miRNA–mRNA interactions forming the ceRNA axes for each disorder

To validate whether the miRNAs identified in the previously constructed ceRNA network were also differentially expressed in MZ twins discordant for psychiatric disorders, we performed sRNA-seq on the same MZ twin cohort (Fig. 1A). Differentially expressed miRNAs (DE-miRNAs) were identified using DESeq2 (*p* < 0.05, |log_2_ FC|> 0.585; Fig. 4A-C). In the BDC cohort, 32 DE-miRNAs were identified (9 upregulated, 23 downregulated); the MDC cohort revealed 33 DE-miRNAs (10 upregulated, 23 downregulated); and the SDC cohort yielded 21 DE-miRNAs, all of downregulated (Supplementary Table S10-S12).

We next intersected the identified DE-miRNAs with those from the previously constructed ceRNA networks to define disease-specific hub ceRNA networks, composed exclusively of DE-miRNAs, DE-mRNAs and DE-lncRNAs. The resulting hub networks in BD consisted of 4 DE-miRNAs, 5 DE-mRNAs, and 3 DE-lncRNAs (Fig. 4D and G); in MDD, 4 DE-miRNAs, 4 DE-mRNAs, and 3 DE-lncRNAs (Fig. 4E and H); and in SCZ, 3 DE-miRNAs, 7 DE-mRNAs, and 3 DE-lncRNAs (Fig. 4F and I, Supplementary Table S13). Cross-disorder comparison revealed hsa-miR-29a-3p as only miRNA shared between MDD and SCZ hub networks. This miRNA was significantly downregulated in both MDD (log_2_FC = -0.608, *p* = 3.96E-4) and SCZ (log_2_FC = -1.457, *p* = 4.97E-2), but not in BD (log_2_FC = -0.32, *p* = 0.762). It participated in distinct ceRNA axes: in MDD, involving the *STX18-AS1*, *HCG18*, and *COL6A6;* and in SCZ, involving *JPX*, *PCBP1-AS1* and *COL6A6*.

**Fig. 5:**
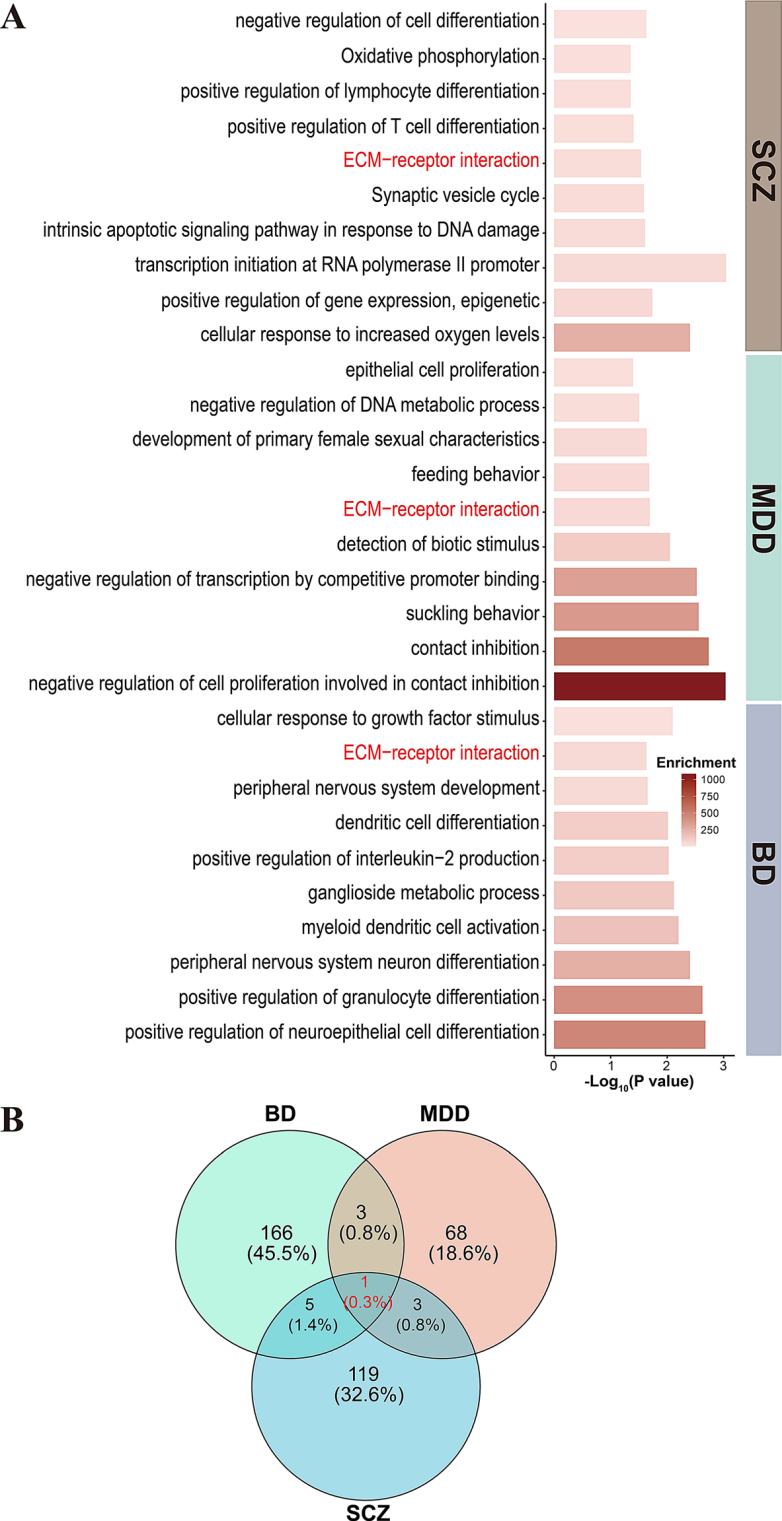

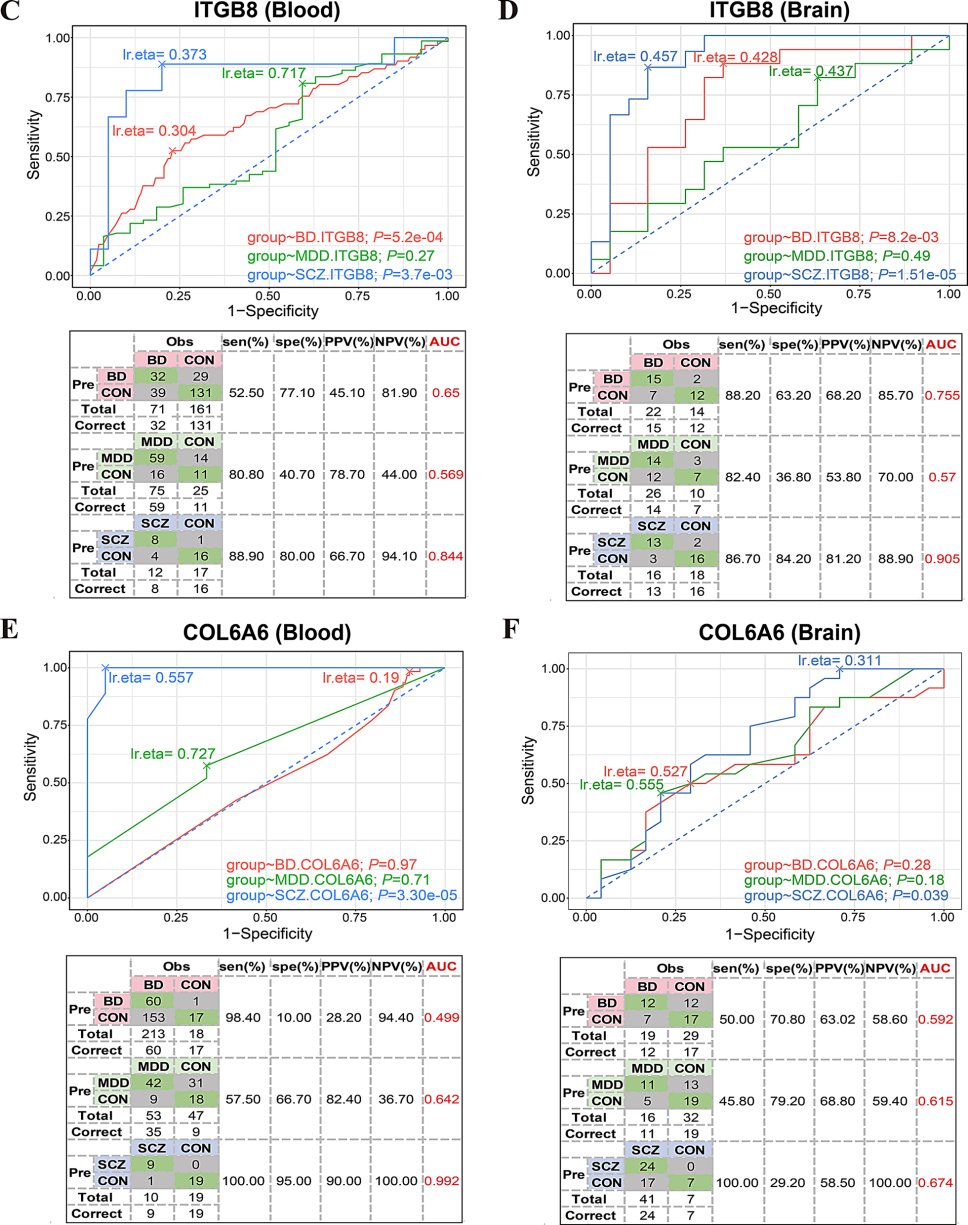
Shared pathway enrichment and predictive evaluation of ceRNA network components in psychiatric disorders. (**A**) Bar plot of significantly enriched biological processes and pathways associated with hub ceRNA network components (DE-mRNAs and DE-lncRNAs targeted by DE-miRNAs) in SCZ, MDD, and BD. (**B**) The Venn diagram illustrates the overlap of significant enriched processes or pathways identified from hub ceRNA network components in BD, MDD, and SCZ. Processes or pathways common to all three disorders are highlighted in red. (**C**) Receiver operating characteristic (ROC) curve assessing the predictive potential of *ITGB8* in blood for BD, MDD, and SCZ. Biological relevance is evaluated using sensitivity, specificity, positive predictive value (PPV), negative predictive value (NPV), optimal fitted value (lr.eta), and area under the curve (AUC). (**D**) ROC curve for *ITGB8* in prefrontal cortex (PFC) region, evaluating its biological relevance in BD, MDD, and SCZ. (**E**) ROC curve for *COL6A6*, shared between MDD and SCZ, evaluated in blood. (**F**) ROC curve for *COL6A6* in the anterior cingulate gyrus (AnCg). Biological relevance metrics (sensitivity, specificity, PPV, NPV, AUC) are shown for each dataset

To investigate the functional relevance of these networks, we performed enrichment analyses using DE-mRNAs and DE-lncRNAs from each hub ceRNA network. Results revealed overlapping functional pathways across the disorders, including extracellular matrix structural constituent conferring tensile strength, and notably, ECM-receptor interaction (Fig. 5A, B and Supplementary Table S14). The ECM-receptor interaction pathway emerged as a convergent signature across all three disorders. Supporting this observation, the ECM-receptor component *COL6A6* was implicated in both the MDD and SCZ ceRNA networks (log2FC = -1.14, *p* = 4.79e-2 in MDD; log2FC = 0.81, *p* = 4.97e-2 in SCZ), while *ITGB8*, another ECM-related gene, was identified in the BD network (log2FC = 1.282, *p* = 0.044). Importantly, *COL6A6* was regulated by hsa-miR-29a-3p in both MDD and SCZ ceRNA networks (Fig. 4H, I), further supporting its roles in shared disease mechanisms.

These findings suggest that dysregulated of the ceRNA-mediated regulatory interactions, particularly involving ECM-receptor signaling, may contribute molecular pathogenesis of psychiatric disorders. They also highlight *COL6A6* and *ITGB8* as promising candidates for future mechanistic and biomarker studies.

### Region-specific biomarkers of ITGB8 and COL6A6 in psychiatric disorders

To evaluate the predictive potential of *ITGB8* and *COL6A6*, key components of the ECM-receptor pathway identified in the hub ceRNA networks, we performed Receiver operating characteristic (ROC) curve analyses using independent transcriptomic datasets from blood and five brain regions (Fig. 1A and Supplementary Table S1). Biomarkers were considered significant if they achieved an area under the curve (AUC) > 0.65 and a *p*-value < 0.05.

*ITGB8*, identified within the hub ceRNA network for BD, showed strong discriminatory power in both BD and SCZ blood datasets (Fig. 5C), In BD, *ITGB8* achieved an AUC of 0.65 (*p* = 5.16e-4), and in SCZ, the AUC was 0.844 (*p* = 3.73e-3). In the brain, *ITGB8* demonstrated robust discriminatory performance in the prefrontal cortex (PFC) for both BD (AUC = 0.755, *p* = 8.17e-03) and SCZ (AUC = 0.905, *p* < 0.001; Fig. 5D and Supplementary Figure S5), although it did not reach significance in the other brain regions examined.

*COL6A6*, identified in the hub ceRNA networks for both MDD and SCZ, exhibited exceptional classification power in SCZ blood data (AUC = 0.992, *p* = 3.30e-05; Fig. 5E). In brain datasets, *COL6A6* showed moderate prediction performance for SCZ in the anterior cingulate gyrus (AnCg) (AUC = 0.674, *p* < 0.001; Fig. 5F), with critical value for MDD (AUC = 0.615, *p* < 0.001).

These findings highlight *ITGB8* as a potential PFC-specific biomarker, exhibiting biological consistency across blood and brain tissues for both BD and SCZ. In contrast, *COL6A6* may serve as a blood- and AnCg-specific marker, particularly for SCZ. The brain region-specific performance of these markers underscores the importance of integrating spatial transcriptomic resolution in biomarker discovery for psychiatric disorders, advancing precision medicine strategies.

## Discussion

In the present study, we constructed a comprehensive integrative analysis of whole-transcriptome and small RNA sequencing data from peripheral blood samples of MZ twins discordant for SCZ, BD or MDD. Our study identified disease-associated DE-RNAs (DE-lncRNAs, DE-mRNAs, and DE-miRNAs), established ceRNA regulatory networks, and explored shared and disorder-specific molecular mechanisms underpinning these psychiatric conditions.

MZ twins discordant for psychiatric disorders represent a uniquely powerful model for uncover non-genetic regulatory influences, as they provide stringent control over genetics, age, sex, familial background, and early environmental exposures, allowing detection of disease-relevant epigenetic differences with improved sensitivity [46]. Leveraging this unique design, we identified *ARPC4-TTLL3* consistently differentially expressed across all three disorders. This naturally occurring read-through transcript involves *ARPC4*, a gene previously implicated in SCZ pathogenesis through altered actin dynamic and oxidative phosphorylation processes [47]. Additionally, we identified a previously uncharacterized non-coding RNA (ENSG00000260927) associated with SCZ, BD, and MDD. This transcript targets the *CSNK2A2* gene, encoding casein kinase 2 (CK2), which is involved in neurodevelopmental processes including neurogenesis, and synaptic plasticity, and memory extinction via the ERK-CREB signaling pathway [48, 49].

By overlapping the co-expression results with the DEGs, we obtained candidate RNAs corresponding to each disease. Functional annotation of these RNAs revealed key insights. In BD, the function of glial cells in the cerebral cortex was emphasized (gliogenesis, cerebral cortex development), while SCZ, processes Wnt signaling pathway and regulation of apoptotic signaling pathway were highlighted. Conversely, MDD emphasized the functions of toll-like receptor signaling pathway, immune response-regulating signaling pathway, and negative regulation of inflammatory response, indicating disease-specific functional pathologies.

By constructing disease-specific ceRNA networks, we identified shared post-transcriptional regulatory interactions among mRNAs, lncRNAs, and miRNAs. Notably, 19 miRNAs were consistently identified across all three disorder-specific networks, suggesting their potential role as shared regulatory hubs influencing psychiatric pathogenesis. Among these, miR-22-3p, miR-29a-3p, miR-29b-3p, miR-29c-3p, miR-330-5p, miR-326, and miR-17-5p have been previously associated with psychiatric disorders through modulation of synaptic structures, neurotransmission, stress responses, and dopaminergic signaling [39–45]. The shared involvement of these miRNAs further emphasizes their critical regulatory roles across psychiatric conditions.

Although no universally dysregulated mRNAs or lncRNAs were identified, pairwise comparisons consistently highlighted the ECM-receptor interaction pathway as a convergent signature across SCZ, BD, and MDD. This is consistent with previous transcriptomic and genetic studies showing that ECM-mediated signaling, particularly integrin-dependent pathways, plays key roles in neuronal development, synaptic plasticity, and oligodendrocyte maturation [50–52]. ECM alterations have also been linked to stress response and immune activation in MDD [50], and in SCZ, rs10786700 deletion reduces *SUFU* expression, impairs neurogenesis, and disrupts ECM-receptor signaling and dendritic spine density [52]. However, prior studies have not examined ECM-receptor disruption at the level of transcriptional regulatory networks.

Our findings extend this literature by suggesting that convergent ECM-related changes may emerge from coordinated transcriptomic alterations rather than shared directionality of individual genes. Within this context, *ITGB8* and *COL6A6* emerged as key ECM-associated candidates. Notably, COL6A6 showed opposite directions of effect—downregulated in MDD but upregulated in SCZ—indicating disease-specific upstream regulatory environments rather than a common mechanism. This is biologically plausible: COL6A6 anchor the basement membrane to underlying connective tissue [53], and increased expression promotes ECM thickening and fibrosis during ageing and pathological remodeling [54, 55], whereas reduced expression weakens ECM integrity and may affect cognitive and synaptic stability [56]. Consistent with these disorder-specific patterns, ROC validation showed significant biological relevance for *ITGB8* in the PFC of BD and for *COL6A6* in the AnCg of SCZ. Together, these findings underscore the importance of ECM-associated pathways in psychiatric pathophysiology, corroborating evidence from genetic, transcriptomic, and microbiota studies implicating ECM dysregulation in psychiatric disorders [52, 57–59].

Despite the valuable insights gained, this study has several limitations. First, the modest sample size inherent to discordant MZ twin studies may limit statistical power, particularly for detecting subtle transcription changes. Additionally, the use of raw p-values and an *r* > 0.2 threshold in our analyses may increase the risk of false positives, reducing confidence in some findings. Larger cohorts, independent replication, and more stringent thresholds are necessary to strengthen these results. Second, detailed information on psychotropic medication use, smoking status, BMI, recent infections, and other blood-related covariates was not systematically collected. While the MZ twins design helps control for genetic background and shared early-life environments, residual confounding, particularly from medication exposure, cannot be fully excluded. Therefore, potential clinical influences on gene expression should be interpreted with caution. Finally, while peripheral blood is a practical and informative window into systemic molecular alterations, it may not fully capture brain-specific transcriptional signatures. Future studies integrating large sample sizes and brain tissue datasets will be important for validating and extending these findings.

In conclusion, this integrative transcriptomic and network-based study highlights the critical roles of non-coding RNA-mediated regulatory interactions and ECM-receptor pathways across psychiatric disorders. Our findings offer mechanistic insights, suggesting shared molecular vulnerabilities underlying these heterogeneous psychiatric conditions. These data advance our understanding of psychiatric pathogenesis and suggest potential biologically relevant biomarkers for precision psychiatry, ultimately contributing to improved diagnosis, treatment stratification, and therapeutic development.

## Supplementary Information

Below is the link to the electronic supplementary material.

Supplementary Figures

Supplementary Tables

## Data Availability

The publicly available data that support the findings of this study are openly available in GEO at [https://www.ncbi.nlm.nih.gov/geo/](https:/www.ncbi.nlm.nih.gov/geo) , reference number GSE46449, GSE98793, GSE27383, GSE124326, GSE247998, GSE263180, GSE53987 and GSE80655. All data generated or analyzed during this study are included in this published article and its supplementary information files or are available from the corresponding author on reasonable request.
