# Supplementary material for: Integrative ceRNA network analysis in monozygotic twins reveals shared and disorder-specific molecular signatures in major psychiatric disorders
Source: BMC Psychiatry. 2026 Feb 14;26:253. doi: 10.1186/s12888-026-07894-5 (PMC13011503; doi:10.1186/s12888-026-07894-5)
Supplement: Supplementary file 1 — Supplementary Figures [file 12888_2026_7894_MOESM1_ESM.docx]

# Supplementary Figures


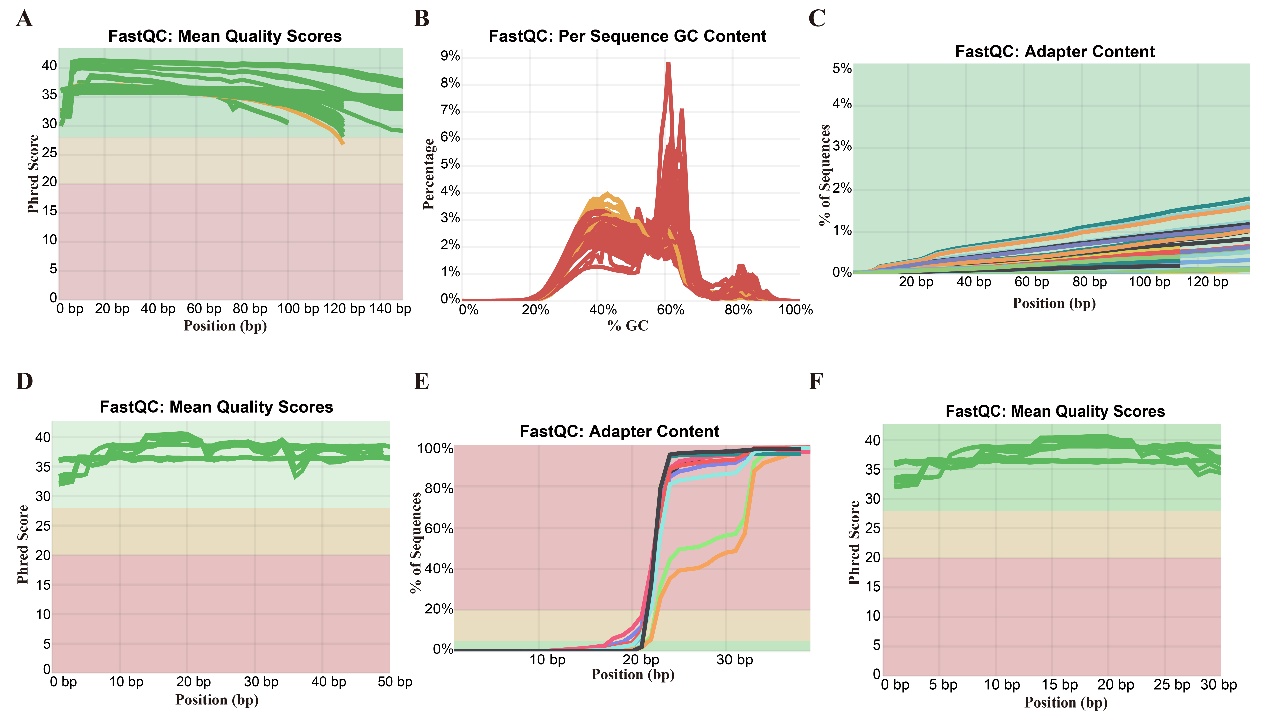


**Figure S1 MultiQC Quality Control Report for RNA-seq and sRNA-seq. (A-C)** This is an RNA-seq quality control report showing mean quality scores. The green line indicates samples that meet quality control requirements. It also shows the GC content and adapter status for each sample. **(D, E)** Small RNA-seq quality control report: Mean Quality Scores and Splice-off Content Prior to Using Trim Galore. **(F)** Mean Quality Scores plot after processing with trim_galore


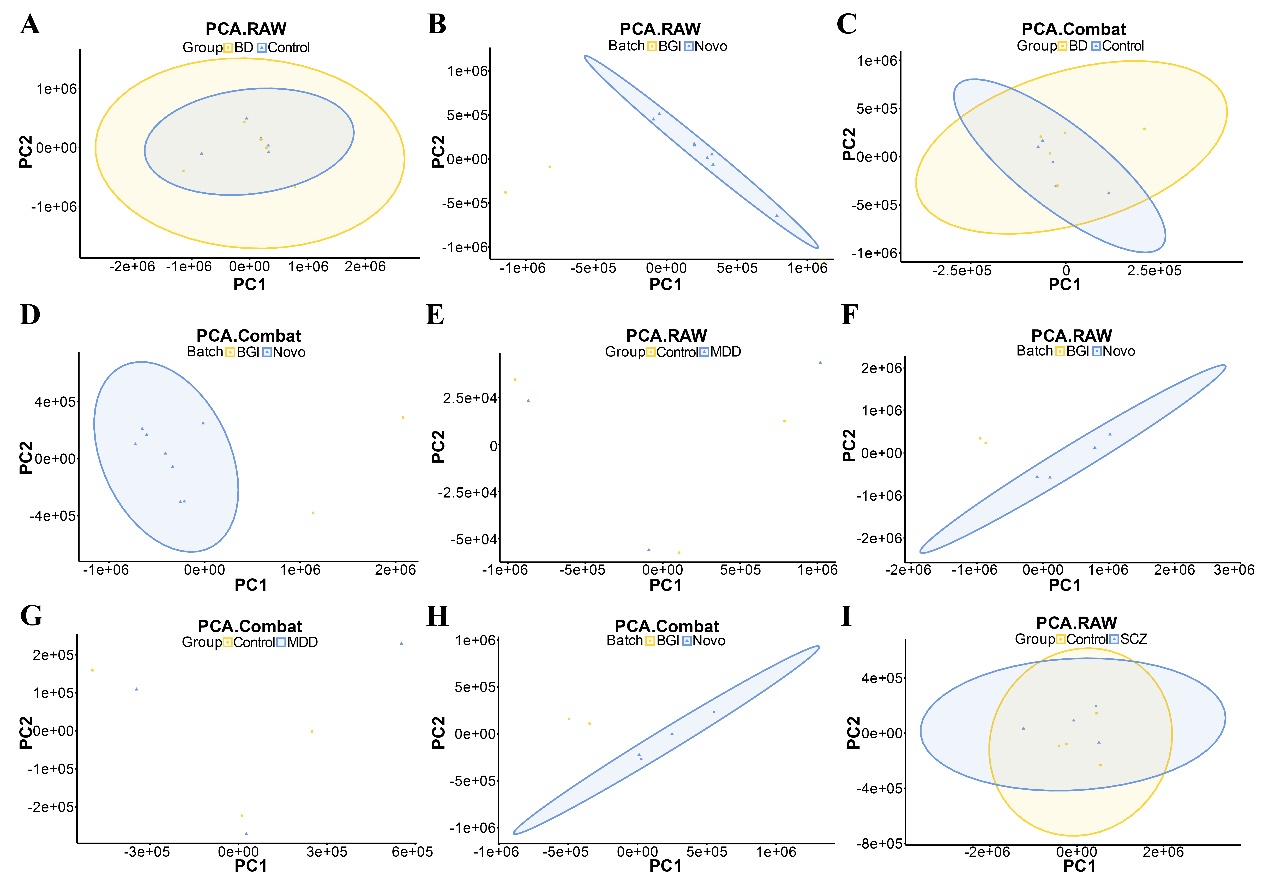


Figure S2 PCA plots of RNA-seq before and after the batch effect remove. PCA plots grouped by disease-control and by batch origin, categorized as PCA before batch correction (PCA.RAW) and PCA after batch correction (PCA.Combat). Ellipses represent 95% confidence intervals.


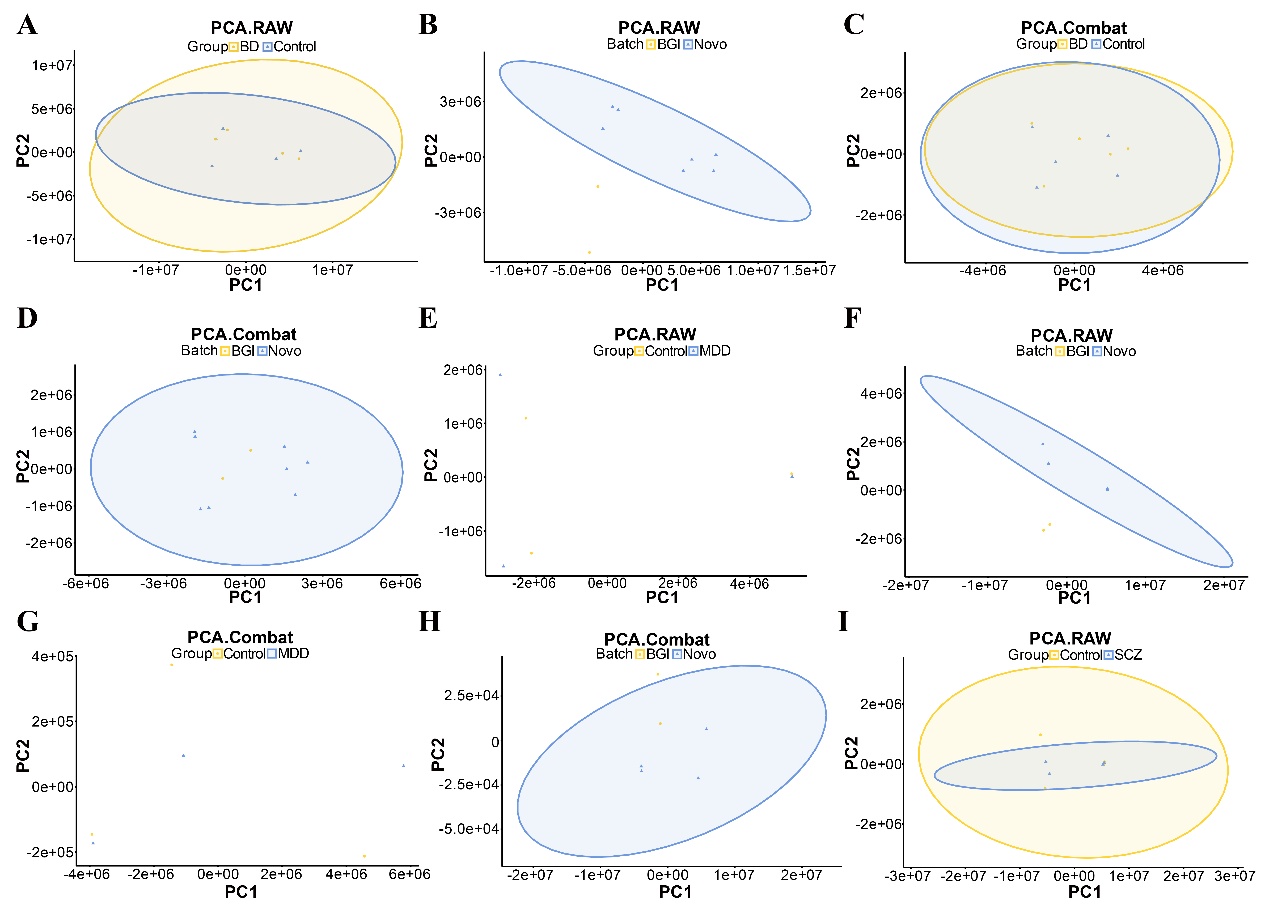


Figure S3 PCA plots of sRNA-seq before and after the batch effect remove. PCA plots grouped by disease-control and by batch origin. The X-axis represents the first principal component (PC1), and the Y-axis represents the second principal component (PC2).


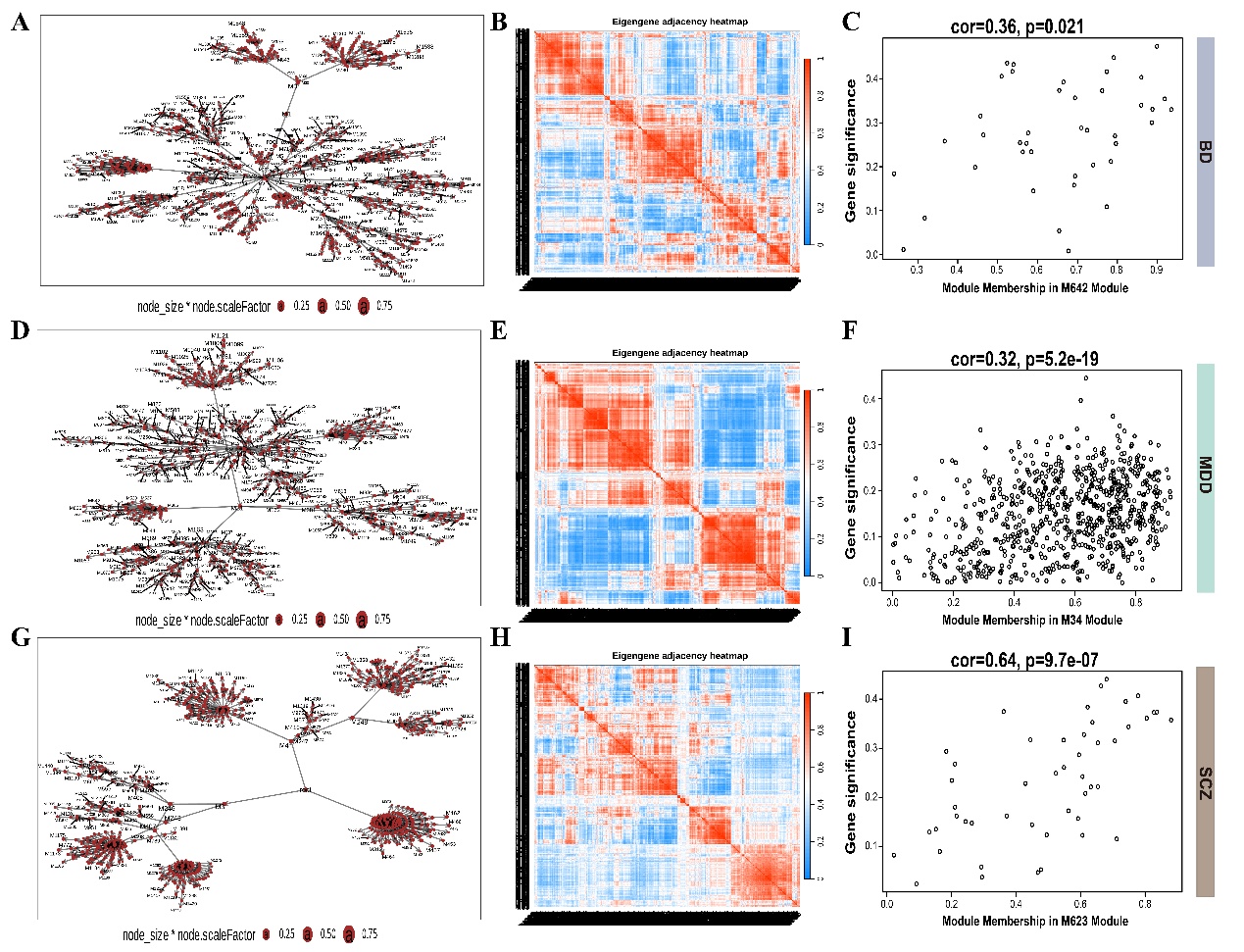


Figure S4 Multiscale Co-expression Module Network Constructed by MEGENA and Module Heatmap in BD, MDD, and SCZ. Network diagrams illustrating the hierarchical structure of co-expression modules identified by MEGENA for BD, MDD, and SCZ. Red nodes represent co-expression modules, with node size proportional to the number of genes contained within each module. Gray arrows indicate hierarchical relationships, with edges directed from parent modules to nested child modules, reflecting multiscale modular organization. The Eigengene adjacency heatmap visualizes the correlations between co-expression modules, ranging from negative (blue) to positive (red) correlations and color-coded accordingly. The scatter plot shows the relationship between how important a gene is in a module (MM) and how much a gene is related to target traits (GS).


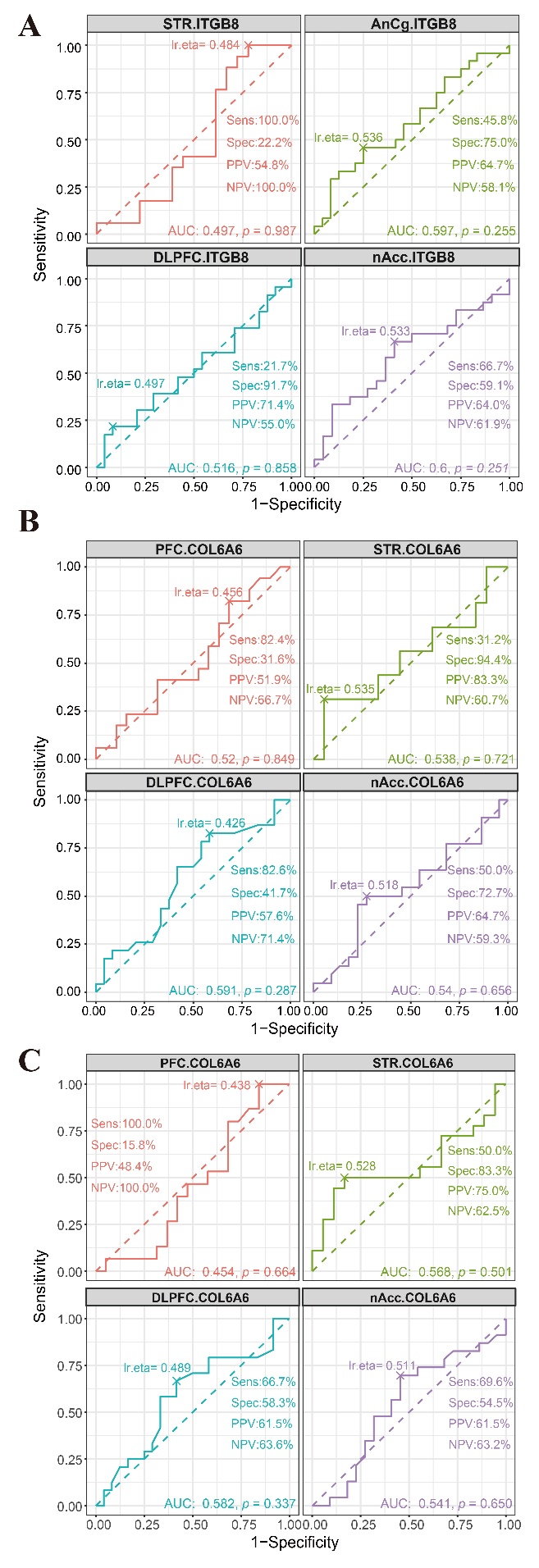


Figure S5 ROC curve analysis for ITGB8 and COL6A6 across four additional brain regions. anterior cingulate gyrus (AnCg), dorsolateral prefrontal cortex (DLPFC), nucleus accumbens (nAcc), and striatum (STR), using independent transcriptomic datasets.
